# Measurement of Free-Form Curved Surfaces Using Laser Triangulation

**DOI:** 10.3390/s18103527

**Published:** 2018-10-18

**Authors:** Zhixu Dong, Xingwei Sun, Weijun Liu, Heran Yang

**Affiliations:** School of Mechanical Engineering, Shenyang University of Technology, Shenyang 110870, China; dong_zhixu@sut.edu.cn (Z.D.); wjliu@sia.cn (W.L.); yangheran@sut.edu.cn (H.Y.)

**Keywords:** laser triangulation, laser spot, free-form curved surface, on-machine measurement

## Abstract

Laser triangulation (LT) is widely used in many fields due to its good stability, high resolution and fast speed. However, the accuracy in these applications suffers from severe constraints on the data acquisition accuracy of LT. To solve this problem, the optical triangulation principle, the object equation of the optical path relationship and the deviation of the laser spot centroid are applied to deduce a mathematical model. Therefore, the image sensor inclination errors can be quantitatively calculated, and the collected data are compensated in real time. Further, a threshold sub-pixel gray-gravity (GG) extraction algorithm is proposed; the gradient function and Gaussian fit algorithm are used to set thresholds to remove the impact of the spot edge noise area on the center location; and polynomial interpolation is employed to enhance the data density of the traditional GG method, thus improving the data acquisition accuracy of LT. Finally, the above methods are applied to on-machine measurement of the American Petroleum Institute (API) thread and the screw rotor, respectively. The experimental results prove that the proposed method can significantly improve the measurement accuracy of free-form curved surfaces using LT and that the improved laser spot center extraction algorithm is more suitable for free-form curved surfaces with smaller curvature and more uniform curvature changes.

## 1. Introduction

In recent years, non-contact optical instruments based on laser triangulation (LT) have been increasingly used in size measurement in the fields of automobiles [1,2], automation [3,4,5], machine tools [6,7], papermaking [8] and construction [9,10], due to their low-cost, high-efficiency and non-invasive features. Especially for measurement of the free-form curved surfaces with regular or irregular shapes, the advantage of LT is more obvious. However, the above applications all equate the accuracy of the commercial sensor itself with that of the entire measuring system and lack effective compensation for the data acquired by LT, resulting in poor measurement results [11].

Many factors can interfere with the data acquisition accuracy of LT, which can be generally divided into three categories: the inherent characteristics of LT, the measurement environment and the surface properties and geometries of the measured object. Furthermore, many scholars have conducted extensive studies on these three aspects. The impact of the inherent characteristics of LT on its data acquisition accuracy can be further divided into two factors: the nonlinearity of the triangulation model and the fluctuation of the laser beam. The problem that the nonlinearity of the triangulation model affects the measurement accuracy of LT is usually solved by using the calibration method. Sun et al. [12] and Zhang et al. [13], considering that the geometric parameter calibration process is tedious, established new data acquisition models respectively based on different methods and proved experimentally that the proposed calibration method is efficient. Yang et al. analyzed the reason for the laser beam dithering in LT and then proposed a method for suppressing the dithering impact, thereby improving the measurement accuracy of LT to a certain degree [14]. With respect to the impact of the measurement environment on the data acquisition accuracy of LT, Alam et al. researched the impact of environmental factors such as temperature, humidity, mechanical vibration and external light on LT. Finally, Alam gave a calibration method to improve the data acquisition accuracy and used dual non-diffracting beams as a light source to enhance the anti-noise performance of laser measurement [15]. Herrmann and Otesteanu, in view of the phenomenon that LT acquires data unstably in the dark or in a strongly-reflective environment, proposed an algorithm for improving the line-detection stability of LT based on the geometric Brownian motion [16]. With respect to the impact of the surface properties and geometries of the measured object on the data acquisition accuracy of LT, Rico et al. proposed an LT-based holographic measurement technique that reduces the measurement error caused by reflectivity, colors and speckle noise [17]. In addition, considering that the inclined plane has a greater impact on the data acquisition accuracy of LT than other factors, Dong et al. built a quantitative inclination error compensation model based on the geometrical relationship between the incidence and receipt of the light centroid, and this model effectively improves the data acquisition accuracy of LT [18]. Furthermore, the inclination angle between the reflected light and the image sensor also has a certain impact on the measurement results during the data acquisition by LT. In spite of that, there are no existing references related to this problem.

The in-depth study of the above LT-based data acquisition accuracy manifests that various influencing factors can lead to the deviation of the spot center on the image sensor, and then the occurrence of measurement errors. Consequently, the rapid and accurate spot center extraction can help enhance the accuracy of LT. To meet the requirements of spot center extraction with high accuracy, fast speed, good robustness and low noise, the sub-pixel center extraction algorithm has attracted more attention. The gray-gravity (GG) method is widely applied to spot center extraction due to its simple operation and fast calculation speed, but it is particularly sensitive to noise, resulting in lower accuracy [19,20]. Additionally, the Steger method is also often used in spot center extraction due to its high robustness and accuracy. However, the process falls in Gaussian iteration, and its computational efficiency is relatively low [21]. Liu et al. [22] and Jiang et al. [23] respectively utilized the graphics processing unit (GPU) algorithm and the field programmable gate array (FPGA) algorithm to improve the Steger method, reducing a certain number of iterations. Even so, the calculation time is still too long to complete the real-time extraction of the laser spot center. In addition to the above methods, Sun et al. put forward a spot center extraction algorithm based on the grey level moment and the smoothing spline algorithm, improving extraction accuracy [24]. Li et al. applied the multi-scale method to spot center extraction and also achieved favorable results [25]. However, these methods are also computationally time-consuming on account of the complex process. To sum up, increasing the noise immunity of the GG method is an effective means to realize the rapid and accurate extraction of the laser spot center.

To solve the above problems, LT error compensation and LT spot center extraction are researched in this paper. Firstly, for LT error compensation, this paper takes advantage of the optical triangulation principle and the geometric relationship of optical paths to establish an image sensor inclination error model and analyzes the effects of various measurement parameters on errors by using the laser displacement sensor (LDS) (KEYENCE LKH080) as the research object. Secondly, for LT spot center extraction, a variable threshold sub-pixel GG extraction algorithm is proposed. In this algorithm, the gradient function and Gaussian fit algorithm are used to set thresholds to eliminate the noise interference. Finally, research results are applied to on-machine measurement of the American Petroleum Institute (API) thread and the screw rotor. The experimental results prove that the above algorithm can substantially improve the measurement accuracy of free-form curved surfaces using LT. Moreover, the comparison between the two sets of on-machine experimental results reveals that the variable threshold sub-pixel GG extraction algorithm is more suitable for free-form curved surfaces with smaller curvature and more uniform curvature changes.

## 2. Methods

**Figure 1:**
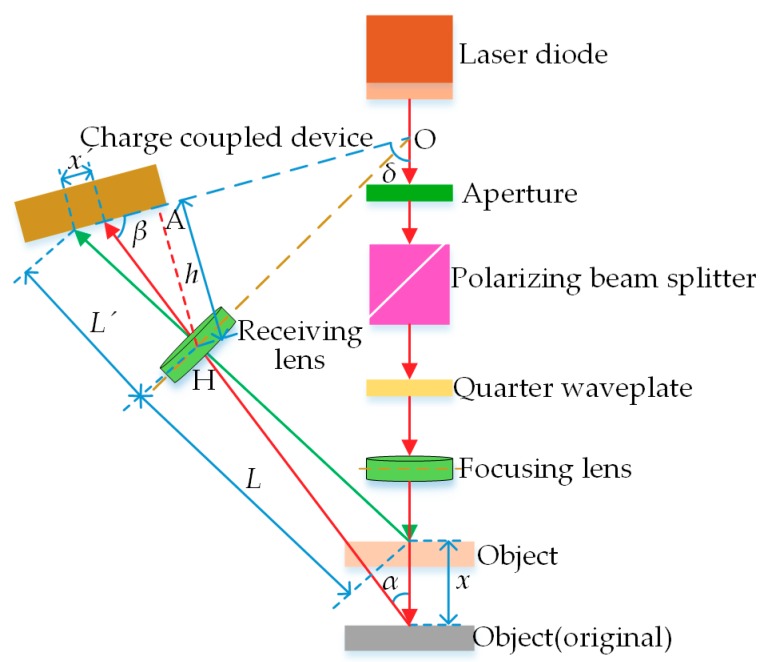
Sketch of the laser triangulation (LT) measurement method.

The LT-based sensor mainly comprises a laser driver, a laser diode, a focusing lens, a receiving lens, an image sensor (charge coupled device (CCD)) and a signal processing circuit. The fundamentals can be seen in Figure 1, where *x* presents the displacement of the measured object plane, *x*′ presents the displacement of the spot image on the CCD, *α* presents the angle between the optical axis of the receiving lens and the laser beam, *β* presents the angle between the optical axis of the receiving lens and the CCD surface, *L* presents the object distance of the receiving lens, *L*′ presents the image distance of the receiving lens, Δ presents the angle between the laser beam and the CCD surface and *h* presents the distance between Focus H of the receiving lens and Point A on the CCD surface. The following equation can be derived in accordance with the theorem of similar triangles:

(1) $$x=\frac{Lx^{\prime }\sin\beta }{L^{\prime }\sin\alpha -x^{\prime }\sin\left(\alpha +\beta \right)}\;$$

Equation (1) is the theoretical equation for the LT measurement principle, which indicates that LT reflects the actual displacement *x* of the measured object by using the image point displacement *x*′ presented on the CCD surface via the receiving lens. To guarantee the data acquisition accuracy in practical applications, it is essential to satisfy both Gauss’s law, which is derived from Snell’s law of refraction, and the Scheimpflug principle, namely the receiving lens and the CCD receiving surface of the image sensor intersect at Point O of the incident beam.

### 2.1. Inclination Error Model for Image Sensors

**Figure 2:**
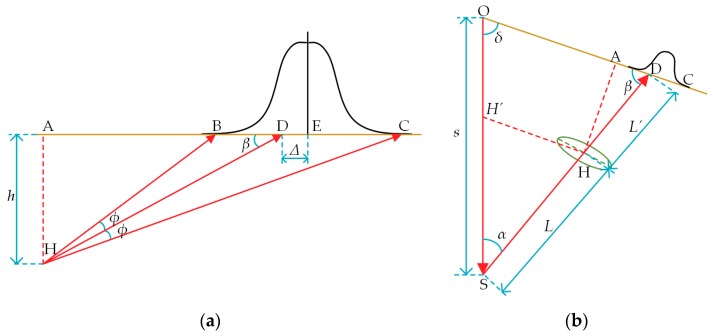
Schematic diagram of generating image sensor inclination errors. (**a**) Geometric relation diagram of the image sensor; (**b**) geometric relation diagram of LT optical paths.

Figure 2 is a schematic diagram of generating image sensor inclination errors, of which Figure 2a is a geometric relation diagram of the image sensor and Figure 2b is a geometric relation diagram of LT optical paths. In Figure 2a, AC denotes the CCD receiving surface, BC denotes the diameter of the reflected light spot received by the CCD, the light intensity of which obeys the Gaussian distribution, Point E denotes the theoretical centroid of the reflected spot, Point D denotes the actual centroid of the reflected spot, *φ* denotes the scattering half angle of the reflected beam, *β* denotes the inclination angle between the reflected light and the CCD receiving surface, Δ denotes the displacement between the theoretical Centroid E and the actual Centroid D and Point H and distance *h* are as mentioned above. It can be seen from Figure 2a that the light beam reflected into the sensor is not perpendicular to the CCD surface, and the inclination angle *β* appears (when and only when the measuring distance is the measuring center of the LT-based sensor, the inclination angle *β* is 0). This causes the spot centroid theoretical Position E detected by the CCD to not coincide with the actual Position D of the spot centroid, and generates the deviation Δ. Accordingly, the error model is deduced according to the following steps:

Firstly, the laser beam scattering rate is defined as:

(2) tanφ=k 

Then, it is obtained that:

(3) AD=hcotβ 

(4) $$\mathrm{AB}=h/\tan\left(\beta +\varphi \right)=h/\frac{\tan\beta +\tan\varphi }{1-\tan\beta \tan\varphi }\;$$

(5) $$\mathrm{AC}=h/\tan\left(\beta -\varphi \right)=h/\frac{\tan\beta -\tan\varphi }{1+\tan\beta \tan\varphi }\;$$

In light of the above equations, it is further obtained that:

(6) $$\mathrm{AE}=\frac{\mathrm{AB}+\mathrm{AC}}{2}=h\frac{\left(1+k^{2}\right)\tan\beta }{\tan^{2}\beta -k^{2}}\;$$

Therefore, the deviation error Δ of the spot center on the image sensor is:

(7) $$\Delta =\mathrm{AE}-\mathrm{AD}=h\frac{k^{2}\left(\tan^{2}\beta +1\right)}{\tan^{3}\beta -k^{2}\tan\beta }\;$$

When Δ is brought into the measuring equation of the direct optical triangulation principle, the image sensor inclination error can be obtained as follows:

(8) $$\Delta_{s}=\frac{L\Delta \sin\beta }{L^{\prime }\sin\alpha -\Delta \sin\left(\alpha +\beta \right)}\;$$

where $\Delta_{s}$ is the image sensor inclination error, the measurement parameters *α*, *β*, *L* and *L*′ vary with the measuring distance *s* and the remainder are the structural design parameters of LT. The change law of each parameter with *s* can be calculated from the geometric relation in Figure 2b.

The relations among *α*, *β* and *s* can be derived upon the theorem of similar triangles and the sine theorem as follows:

(9) $$\beta =\arctan\frac{s\sin\delta }{\mathrm{OD}-s\cos\delta }\;$$

(10) α=π−β−δ 

In light of the theorem of similar triangles and the cosine theorem, the relations among *L*, *L*′ and *s* can be derived as follows:

(11) $$L=\sqrt{{\left(s-OH^{\text{′}}\right)}^{2}+{HH^{\text{′}}}^{2}-2\left(s-OH^{\text{′}}\right)HH^{\text{′}}\cos\delta }\;$$

(12) $$L^{\prime }=\frac{OH^{\text{′}}}{s-OH^{\text{′}}}L\;$$

**Figure 3:**
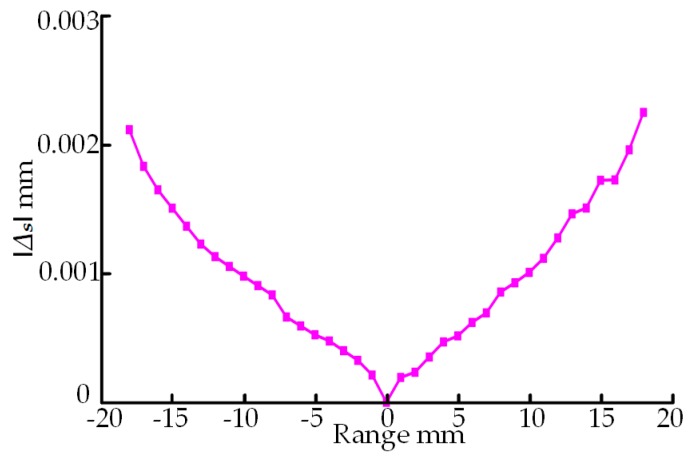
Relationship between $\Delta_{s}$ and *s*.

Through analyzing the above equations, it can be concluded that the inclination error $\Delta_{s}$ is only related to the measuring distance *s*. To clarify the relationship between $\Delta_{s}$ and *s* and prepare for the follow-up experiments, KEYENCE LKH080 LDS is taken as an example here (see Table 1 for its main technical parameters). LDS inclination errors are calculated at 1-mm intervals within its measuring range, and the changes of $\Delta_{s}$ with *s* are shown in Figure 3. It can be found from Figure 3 that the change law of $\Delta_{s}$ with *s* is that within the range, the image sensor inclination error is proportional to the measuring distance and is in the same direction as the displacement of the object plane; when the measuring distance is the half measuring range position, the image sensor inclination error is zero.

The above results reflect the quantitative model for image sensor inclination errors constructed in this paper. Although some numerical values have been approximated during the equation derivation, the model still increases the data acquisition accuracy of LT to some extent, engineeringly.

### 2.2. LT Spot Center Extraction Algorithm

To achieve the high-accuracy extraction of the laser center, this paper proposes the variable threshold sub-pixel GG extraction algorithm. The improved algorithm uses the gradient threshold method and Gaussian fit algorithm to remove the interference of spot edge noise area and applies polynomial interpolation to reinforce the data density, thus remarkably heightening the positioning accuracy of the spot center.

Optical images usually represent pixels by using a combination of three primary colors of red, green and blue, and the color values of each pixel need to be calculated by a two-dimensional matrix of three color components (R value, G value and B value). The LDS used in this paper is a red laser, and the G value and B value of its spot image are both 0. For this reason, the pixel color can be represented only by the R value. Set the R value function of the spot as f(x,y), then the gradient function at point (x,y) is defined as:

(13) $$G\left[f\left(x,y\right)\right]={\left[\frac{\partial f}{\partial x},\frac{\partial f}{\partial y}\right]}^{T}\;$$

The gradient value is the increment of unit distances in the direction of the maximum changing rate of f(x,y). Given that the laser spot can be approximately circular, the spot image gradient along the directions of f(x,y) and f(x+1,y+1), as well as f(x+1,y) and f(x,y+1) is the largest. Many experiments are devoted to studying the vertical incidence and oblique incidence of laser beams, and the following gradient operator is obtained after the comparative analysis of the experimental results:

(14) $$G\left[f\left(x,y\right)\right]=\left|\sqrt{3}f\left(x+1,y+1\right)-f\left(x,y\right)\right|+\left|\sqrt{3}f\left(x+1,y\right)-f\left(x,y+1\right)\right|\;$$

The maximum R value gradient function of the spot can be found by scanning the whole spot image and conducting gradient operation of the R value of each pixel. The $k_{1}\left(k_{1}<1\right)$-fold maximum gradient value is used as the identification threshold value, of which the *k*_1_ value can be adjusted according to the positioning accuracy degree. Furthermore, the *k*_1_ value is taken as follows: The value is set at 0.1 intervals within (0, 1), and the measurement index should not only effectively eliminate the edge noise area, but also retain the sufficient effective area for the next threshold determination. KEYENCE LKH080 LDS spots used in this paper are calculated by using the gradient operator in Equation (14), with the result that the effect is optimum when *k*_1_ = 0.2. After scanning the spot image over again, the area surrounded by pixels whose R gradient value is equal to the identification threshold value is named the primary threshold area S_1_, the image in S_1_ is retained and transformed into a grayscale image, with the gray function denoted as g(x,y), and the final high-level threshold area S_2_ is determined in S_1_.

As described in Section 2.1, the intensity of laser light received by the image sensor follows the Gaussian distribution, and its Gaussian function is denoted as:

(15) $$F\left(x,y\right)=\frac{1}{2\pi \sigma^{2}}\exp-\left[\frac{{\left(x-x_{0}\right)}^{2}}{2\sigma_{x}^{2}}+\frac{{\left(y-y_{0}\right)}^{2}}{2\sigma_{y}^{2}}\right]\;$$

Let the fit equation set of points in the primary threshold area S_1_ be:

(16) B=AK 

where **B** is the light intensity matrix in S_1_, **A** is the position matrix in S_1_ and **K** is the undetermined coefficient matrix. Since:

(17) $$\ln F\left(x,y\right)=\ln\frac{1}{2\pi \sigma^{2}}-\left[\frac{{\left(x-x_{0}\right)}^{2}}{2\sigma_{x}^{2}}+\frac{{\left(y-y_{0}\right)}^{2}}{2\sigma_{y}^{2}}\right]\;$$

and the *i*-th row of matrix **A** is:

(18) $$a_{i}=\left[x_{i}^{2},y_{i}^{2},x_{i},y_{i},1\right]\;$$

then the undetermined coefficient matrix **K** is:

(19) $$K^{\prime }=\left[\frac{-1}{2k_{1}},\frac{-1}{2k_{2}},\frac{x_{t}}{k_{1}},\frac{y_{t}}{k_{2}},-k_{0}\right]\;$$

where $\left(x_{t},y_{t}\right)$ is the extreme point coordinate of the Gaussian function. The least squares method is used for fitting, so as to minimize the norm of residual vector **E**.

(20) $${\left\|E\right\|}_{2}={\left\|\ln g\left(x,y\right)-B\right\|}_{2}\;$$

The extreme value point $\left(x_{t},y_{t}\right)$ of the fit function is robustly solved by using the above equation, and the gray value at this point is recorded as *I*. Subsequently, the identification threshold is set, with *k*_2_ times *I* as the threshold, and the determination method of the *k*_2_ value is exactly the same as that of the *k*_1_ value, with *k*_2_ = 0.5 given herein. The area where the gray value is greater than the mark threshold *k*_2_*I* is defined as the high-level threshold area S_2_, and the gray value in S_2_ is processed by the Gaussian filters. Considering the large-scale data collected by LT and the strict requirements for real-time data processing, we choose the polynomial interpolation that can better balance the calculation accuracy and the calculation efficiency to densify the filtered spot image. In comparison to polynomial interpolation, spline interpolation has better calculation accuracy, but lower calculation efficiency in the case of a large amount of data and high interpolation density. Additionally, the noise of the spot image is effectively reduced after the variable threshold processing, and hence, polynomial interpolation can meet the accuracy requirements. Therefore, we perform polynomial fit interpolation densification on the filtered spot image at a pitch of 0.1 pixels, and the coordinates of the spot center are obtained by using the GG method for the densified sub-pixel points upon the following equations:

(21) $$x_{0}=\sum_{\left(x,y\right)\in S_{2}}xg\left(x,y\right)/\sum_{\left(x,y\right)\in S_{2}}g\left(x,y\right)\;$$

(22) $$y_{0}=\sum_{\left(x,y\right)\in S_{2}}yg\left(x,y\right)/\sum_{\left(x,y\right)\in S_{2}}g\left(x,y\right)\;$$

**Figure 4:**
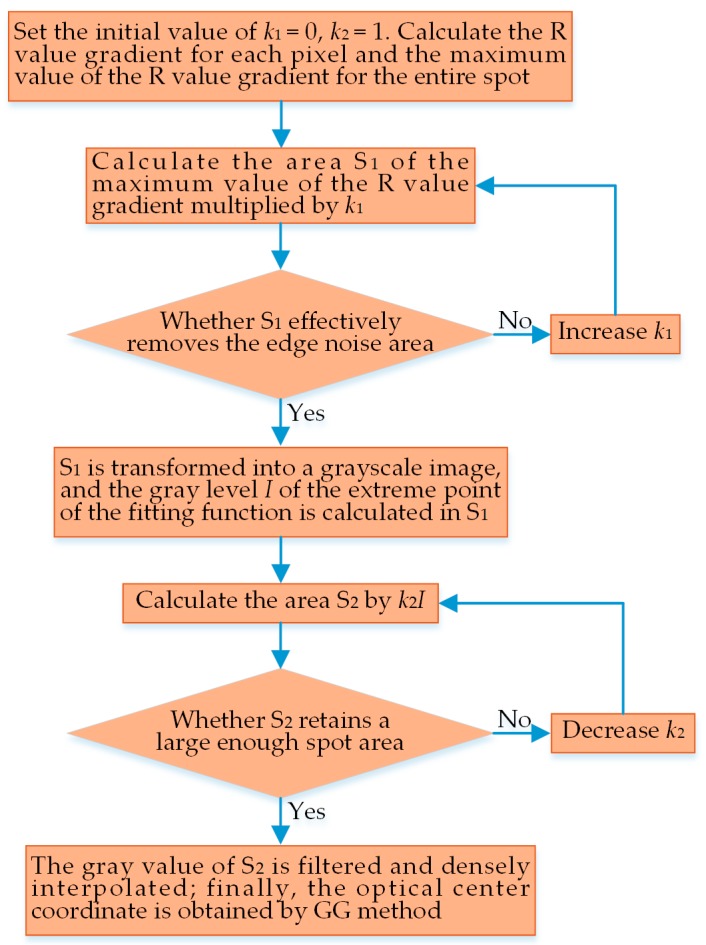
Flowchart of the variable threshold sub-pixel gray-gravity (GG) extraction algorithm.

The length of each pixel of the CCD image sensor used in this paper is 10 μm. When the measurement sensitivity (the ratio of the displacement of the measured surface to the displacement of the spot center detected on the image sensor) reaches the maximum value of 26.86, the measurement error of the imaging spot center can reach 1 pixel, and the measurement error of the measured surface can reach 0.269 mm. It can thus be found that the interpolation and densification of pixel points can effectively increase the positioning accuracy of the spot center. The flowchart of the variable threshold sub-pixel GG extraction algorithm proposed in this section is shown in Figure 4.

## 3. Results

### 3.1. On-Machine Measurement Experiment for API Threads

This section applies the above methods to on-machine measurement of API thread parameters. API threads are mainly used for the conjunction between drill rods in the oil and gas drilling industry and play a role in transmitting the huge torque required for drilling from the wellhead to the bottom-hole bit. As this kind of thread bears large torque and high sealing requirements, it is subject to extremely strict requirements for its contour parameter accuracy. However, the contour parameters are measured by using the monomial parameters gauge (MPG) in every current oil field service station. The main contour parameters of the API thread encompass pitch *p*, thread height *h* and half of thread angle *α*/2.

**Figure 5:**
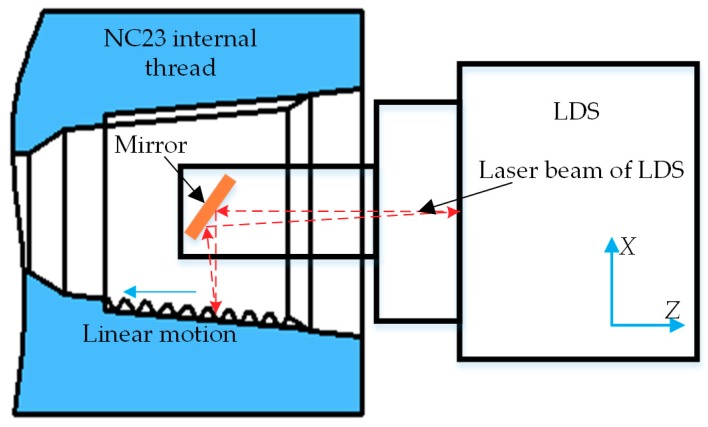
Data acquisition scheme for API thread axial sections.

The minimum internal thread to be measured was the NC23 (number connection, NC) type, and its maximum diameter was only 66.97 mm, while the shell of LKH080 LDS used in this paper was 100 mm long and could not be extended into the interior for measurement, so the mirror panel was used for auxiliary measurement. Through studying various non-contact measurement devices for thread parameters, it can be obtained that each and every measurement device took the thread axial section as the measurement object and then calculated the mathematical relationship between the thread parameters to be measured and the measurement data. Accordingly, the contour data acquisition of one certain API thread axial section was the core task throughout the measurement. The data acquisition scheme for API thread axial sections is shown in Figure 5.

**Figure 6:**
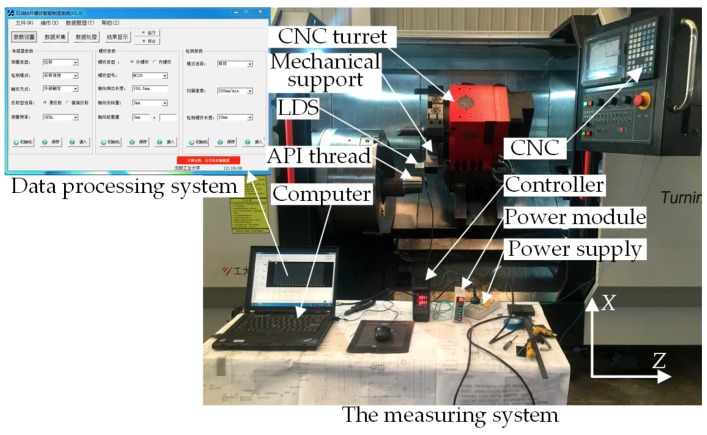
Experimental devices for on-machine measurement of American Petroleum Institute (API) threads.

On the SCK230 CNC lathe, we experimented with on-machine measurement of the processed NC50 API external thread typically used in oil drilling, of which the axial length was 11 mm, as shown in Figure 6. The on-machine measurement experiment was comprised of three components: a measuring system, a CNC system and a data processing system, and mainly included an LDS, a controller and data processing software. Among that, the data processing software, as the kernel of the experiment, owned a rich source of functions, such as measurement parameter setting, data collection based on the variable threshold sub-pixel GG extraction algorithm, data compensation based on the image sensor inclination error model, data reconstruction, parameter calculation and quality judgment of thread processing. The computer equipped with the software was configured with an Intel Core i7 processor with a 64-bit operating system, 3.8-GHz operation speed and 8-GB memory. The LDS used was consistent with the above, for which the specific parameters will not be repeated. The CNC lathe featured three CNC axes, i.e., a radial feed axis *X*, an axial feed axis *Z* and a spindle, for which the *Z*-axial positioning accuracy was 0.01 mm/500 mm and the straightness of axial rail was 0.05 mm/1000 mm. The CNC system of SCK230 was Mitsubishi M70.

**Figure 7:**
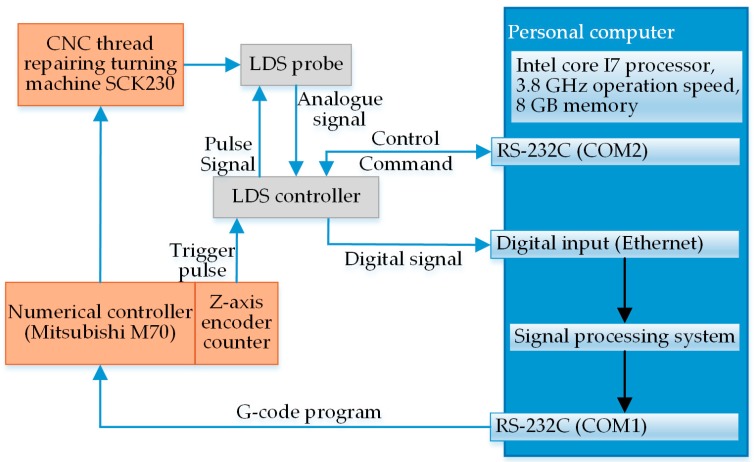
Signal flowchart of the on-machine measurement experiment for API threads.

The processes of the on-machine measurement experiment for the NC50 thread are detailed as follows: The measurement parameters in the data processing system were set, such as the encoder trigger frequency, measurement speed, sensor’s initial position, measured thread type and measured thread length, and then, these parameters were synchronized to the CNC system and controller via different RS232 serial ports. The sensor was furnished with an external signal input that was mounted on the NC turret via a mechanical support. With the movement of the NC turret at a given measurement speed driven by the servo motor, an impulse signal of a certain frequency was transmitted to the input by the *Z*-axis encoder via the controller. Triggered by the valid impulse edge, the sensor synchronously output the measured analog signal to the controller through Ethernet. After one scan was finished by the sensor, the coordinates of the thread axial sections were acquired and then compensated and reconstructed by the data processing system. Further, the required thread parameters were calculated upon the extracted feature points, determining the processing quality. Finally, the results were communicated to the CNC system via RS232 serial ports, and lathing was conducted on the unqualified workpiece for correction as per the given cutter compensation, thus accomplishing the closed loop process of the on-machine measurement. Figure 7 presents a signal flowchart of the on-machine measurement experiment.

**Figure 8:**
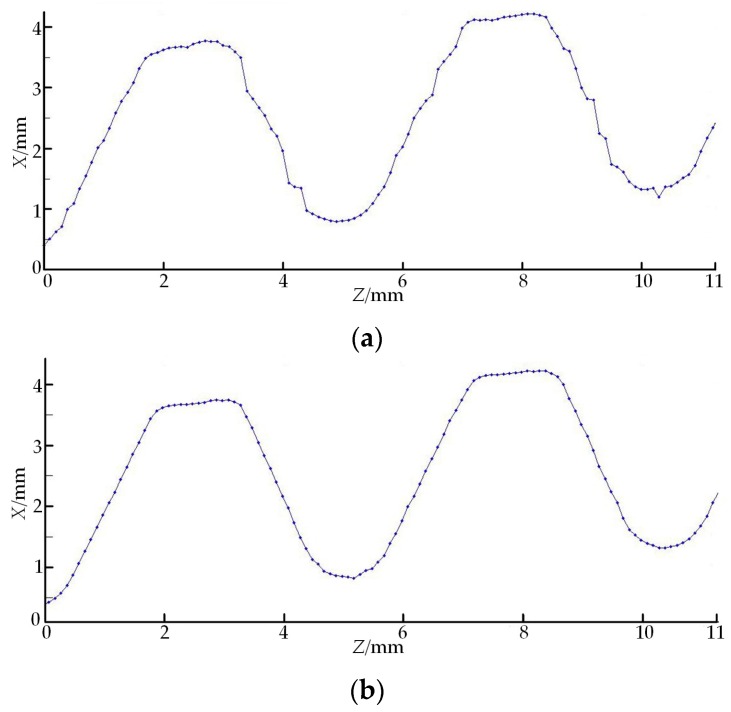
LT contour data obtained by applying different spot center extraction algorithms. (**a**) Traditional GG method; (**b**) variable threshold sub-pixel GG method.

To compare the advantages of the variable threshold sub-pixel GG extraction algorithm proposed in this paper with the traditional GG method, this section utilizes the above two methods to scan the same axial section of the NC50 thread once, respectively, with the results shown in Figure 8. Figure 8a is the contour obtained by using the traditional GG method, and Figure 8b is the contour obtained by using the improved algorithm.

**Figure 9:**
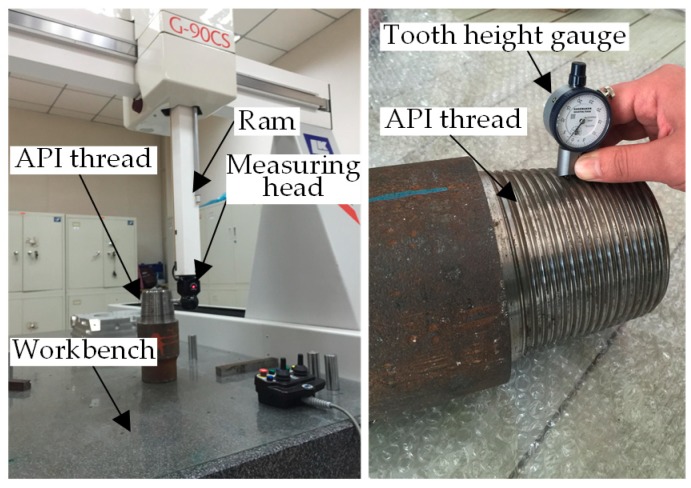
Comparative experiment for measuring API threads.

To verify the application effect of the proposed LT data acquisition method in thread curved surface measurement, firstly, the axial section of the measured API thread was marked and the measurement position and measurement phase of the mark were recorded. Then, the comparative measurement of axial sections was experimentalized by using the coordinate measuring machine (CMM) and MPG, respectively, as shown in Figure 9. The comparative experiment used G90CS CMM manufactured by LK Metrology UK Ltd. (Derby, UK), for which the measuring strokes of each axis were 1500 mm for the *X*-axis, 1000 mm for the *Y*-axis and 800 mm for the *Z*-axis, the maximum permissible error of extent (MPEE) was (2 + L/300) μm, the maximum permissible error of probing (MPEP) was 3.5 μm, the resolution was 0.1 μm, the guaranteed lab temperature was 20 ± 2 °C, the space gradient of temperature was 1 °C/m^3^ and the relative humidity was 40–70%. All the indicators conformed to the requirements of ISO10360-2. The processes of measuring the API thread by using CMM were as follows: As the drilling pipe with the API thread on both ends was approximately 10 m in length and 300 kg in weight, it was impossible to acquire the thread contour data by using CMM. Before measurement, the API threaded joint was necessarily cut off with a saw machine, and the section was accurately machined with a grinder. The API thread was placed on the workbench of CMM with the ground surface as the reference surface. CMM was equipped with a special measuring software for taper thread surfaces. The software could automatically fulfill the probe calibration upon the diameter and contact direction of the selected needle tip. At the time of measurement, firstly, the computer-aided design (CAD) model for the measured thread was imported into the software with the measurement mode of closed linear scan selected. Subsequently, the scanning starting point and scanning direction point were determined on the CAD model. Finally, the scanning control mode, step length and single point were selected, thus accomplishing the measurement of the API thread using CMM. The LDS data fit method was also used to fit the data collected by CMM and calculate the corresponding parameters.

The API thread parameters obtained by the three measurement methods along the same measuring path are shown in Table 2. To compare the effectiveness of the two proposed LT measurement accuracy improvement methods, the thread parameters obtained by the on-machine measurement using LDS were divided into three groups: OMM 1, OMM 2 and OMM 3. Among these, OMM 1 referred to the thread parameters obtained by carrying out direct fit calculation without any compensation for the thread axial section data collected by LDS, OMM 2 referred to the thread parameters obtained by carrying out fit calculation of the thread axial section data collected by LDS, which were compensated by using the image sensor inclination error model, and OMM 3 referred to the thread parameters obtained by carrying out fit calculation of the thread axial section data collected by LDS, which were compensated by using the image sensor inclination error compensation model and processed by using the variable threshold sub-pixel GG extraction algorithm. Moreover, the results in Table 2 are all the average of 10 measurements, and the nominal value and tolerance zone of each parameter of the NC50 thread are also given in the table.

Furthermore, to ensure the integrity of measurement results by OMM 3, the evaluation of measurement uncertainty should be made against thread height *h*, half of the thread angle *α*/2 and pitch *p*. The main factors affecting the measurement uncertainty of thread height *h* are: LDS resolution and linearity errors of LDS, Z-axial positioning accuracy of machine tools and straightness of the axial rail, environmental temperature changes and measurement repeatability. Among these, the measurement uncertainty caused by measurement repeatability was calculated by using the Type A evaluation method, while the measurement uncertainty caused by other factors was calculated by using the Type B evaluation method. These influential factors are analyzed in detail below.

Table 1 shows that LDS resolution can reach 0.1 μm, that is the measurement uncertainty introduced by sensor resolution *u_h_*_1_ = 0.1 μm. Furthermore, it shows that the linearity error of LDS was ±0.02% FS, while the nominal value of the NC50 thread height was 3.095 mm. The error conformed to a uniform distribution, and if coverage factor $k=\sqrt{3}$ was taken, the measurement uncertainty component *u_h_*_2_ generated by the linearity error of LDS within the measurement range is:

(23) $$u_{h}{}_{2}=\frac{0.02\%F.S.}{k}=\frac{0.02\%\times 3.095}{\sqrt{3}}=0.36\;\mu m\;$$

The *Z*-axial positioning accuracy of SCK230 was 0.01 mm/500 mm, and the resulting errors obeyed a uniform distribution. If $k=\sqrt{3}$ was taken and the measured thread was 11 mm long, then the measurement uncertainty component *u_h_*_3_ introduced by the *Z*-axial positioning accuracy of the machine tool is:

(24) uh3=0.01500×113=0.13 μm 

Similarly, the axial rail straightness of SCK230 was 0.05 mm/1000 mm, and the measurement uncertainty component *u_h_*_4_ introduced by itself was 0.32 μm.

When the room temperature *t* was 21.3 °C, 10 measurements of API threads were implemented by using OMM 3 in a short time, and the measurement uncertainty component caused by the environmental temperature change *u_h_*_5_ is:

(25) $$u_{h5}=\alpha h_{d}\left(t-20\right)\;$$

where *α* denotes the coefficient of linear expansion of materials, with its value taken as *α* = 1.1 × 10^−6^/°C and *h_d_* denotes the nominal value 3.095 mm for the NC50 thread height, then *u_h_*_5_ = 0.004 μm.

The Type A evaluation method was applied to the measurement uncertainty *u*_h5_ caused by the measurement repeatability of thread height, and its standard deviation was calculated by using Bessel’s formula:

(26) $$s_{x}=\sqrt{\frac{\sum_{i=1}^{n}\left(x_{i}-\bar{x}\right)}{n-1}}$$

where *n* denotes the number of repeated measurements, *x_i_* denotes each measurement result and $\bar{x}$ denotes the average of *n* measurements. Based on the 10 measurements of thread height obtained by using OMM 3 in Table 2, the standard deviation of thread height *s_h_* = 0.0015 μm was calculated, and the measurement uncertainty introduced is:

(27) $$u_{h6}=\frac{s_{h}}{\sqrt{n}}=0.0004\;\mu m\;$$

As the above measurement uncertainty components are mutually independent, the combined uncertainty *u_h_* of thread height *h* by OMM 3 was calculated by using the square root of the sum of squares:

(28) $$u_{h}=\sqrt{u_{h1}^{2}+u_{h2}^{2}+u_{h3}^{2}+u_{h4}^{2}+u_{h5}^{2}+u_{h6}^{2}}=0.51\;\mu m\;$$

Based on the method for calculating the measurement uncertainty of thread height *h*, the measurement uncertainty of pitch *p* and half of thread angle *α*/2 were evaluated. The main factors affecting the measurement uncertainty of pitch *p* in OMM 3 were LDS resolution, *Z*-axial positioning accuracy of the machine tool and the straightness of the axial rail, environmental temperature changes and measurement repeatability. In accordance with the above calculation method, the standard deviation of 10 measurements of pitch *p* was 0.0012 μm, and the combined uncertainty *u_p_* = 0.40 μm. Furthermore, the main factors affecting the measurement uncertainty of half of thread angle *α*/2 in OMM 3 were LDS resolution and linearity errors, *Z*-axial positioning accuracy of the machine tool and the straightness of the axial rail, environmental temperature changes and measurement repeatability. In accordance with the above calculation method, the standard deviation of 10 measurements of half of thread angle *α*/2 was 0.91′ and the combined uncertainty was *u_α_*_/2_ = 2.76′. Furthermore, the uncertainly values of each thread parameter obtained by using OMM 3 are listed in Table 2.

### 3.2. On-Machine Measurement Experiment for Screw Rotors

This section applies the LT data acquisition method in Section 2 to on-machine measurement of screw rotor contour parameters. With the development of the manufacturing technology of complex spiral curved surfaces, the spiral equipment has extensive applications in many sectors such as petroleum, machinery and pro-environment. Additionally, the screw rotor is the key power-generating part of this kind of equipment, and its contour accuracy is directly related to the mechanical property and service life of the whole spiral equipment. However, the MPG like a micrometer is often used in engineering to perform manual on-machine measurement, which results in low measurement accuracy and efficiency. Therefore, an LDS is used here to measure the curved surface contour of a screw rotor, which helps increase machining accuracy and efficiency.

**Figure 10:**
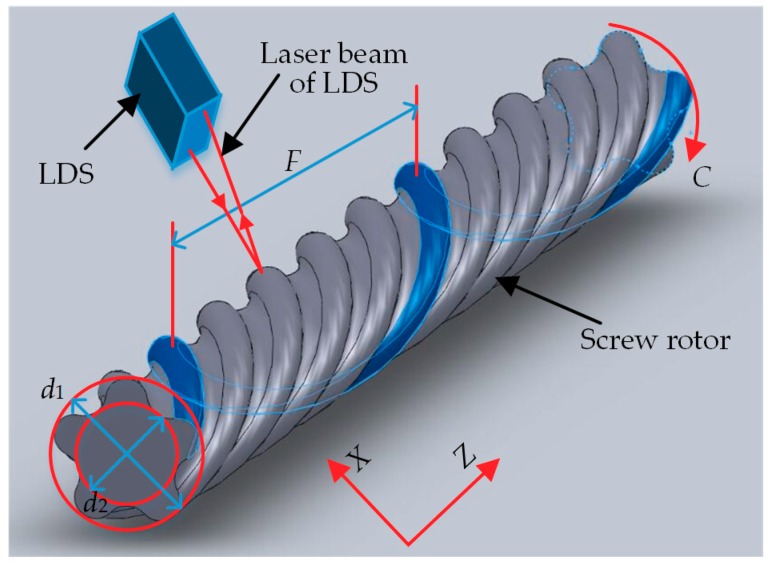
3D model for the screw rotor and schematic diagram of its measuring scheme.

The spiral curved surface of the screw rotor is formed by the motion of the rotor generatrix (cross-sectional contour) along the spiral line, with the diameter scope from 40 mm–200 mm and the length scope from 1000 mm–8000 mm. The 3D model for the 165-type screw rotor with the thread number *n* = 5 commonly used in engineering is shown in Figure 10. The main contour parameters encompass major diameter *d*_1_, minor diameter *d*_2_ and lead *F*. Lead *F* is the length of one thread on the generatrix moving along the spiral line for one cycle, and pitch *w* = *F*/*n* functions as the alternative measurement parameter for the lead. Meanwhile, Figure 10 provides a measurement scheme for the contour parameters of the screw rotor. In the course of measuring major diameter *d*_1_ and minor diameter *d*_2_, LDS keeps still, and the screw rotor rotates one revolution along the *C*-axis. In the course of measuring pitch *w*, the screw rotor keeps still, and LDS moves parallel to the axial centerline of the screw rotor along the *Z*-axis.

**Figure 11:**
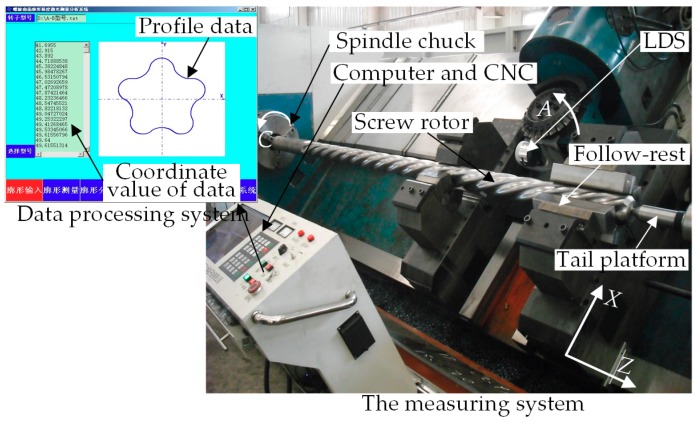
Experimental devices for the on-machine measurement of screw rotors.

In accordance with the manual measurement method (only the major diameter and minor diameter of one cross-section to be measured within one pitch), the on-machine measurement experiment for the finished five-head 165-type screw rotor was performed by the LXK300X CNC spiral groove milling machine, for which the pitch measurement length was 140 mm, as shown in Figure 11. Likewise, the on-machine measurement experiment also consisted of three components: a measuring system, a CNC system and a data processing system. As the kernel of the whole experiment, the data processing system had the following functionality: measurement parameter setting, data acquisition based on the variable threshold sub-pixel GG extraction algorithm, data compensation based on the image sensor inclination error model, parameter calculation and determination of screw processing quality. The computer configuration and LDS model used were consistent with those in Section 3.1. The milling machine was equipped with three NC axes: a radial feed axis *X*, an axial feed axis *Z* and a spindle *C*, for which the *Z*-axis positioning accuracy was 0.01 mm/500 mm and the straightness of the axial rail was 0.05 mm/1000 mm. The CNC used in LXK300X was SINUMERIK 828D.

**Figure 12:**
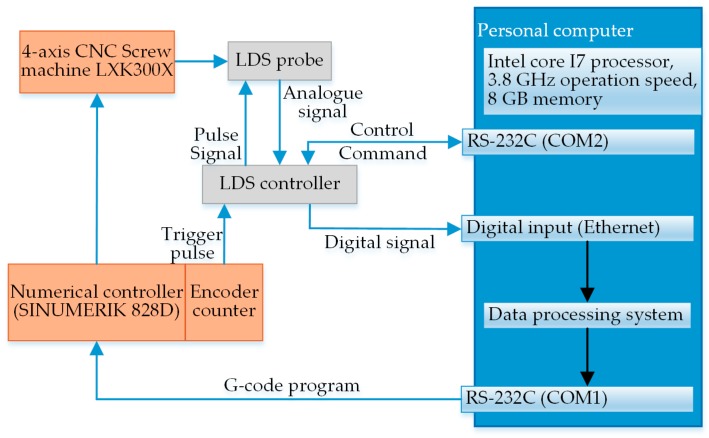
Signal flowchart of the on-machine measurement experiment for screw rotors.

The experimental flow of the on-machine measurement of screw rotors is detailed below. Before the experiment started officially, the measurement parameters were set up in the data processing system, such as the trigger frequency of *C*-axis and *Z*-axis encoders, workpiece rotation speed, sensor moving speed, measured thread type and measured thread length. Then, these parameters were synchronized to the numerical control system and controller via different RS232 serial ports. While measuring major diameter *d*_1_, minor diameter *d*_2_ and pitch *w*, the *C*-axis rotary encoder and *Z*-axis ball screw encoder were respectively transmitted to the external signal input of the sensor upon the aforesaid measuring scheme. At the time of the servo motor driving, an impulse signal of a certain frequency was transmitted by the encoder to the input through the controller; the synchronous output of the measured analog signal was performed by the sensor to the controller via Ethernet triggered by the valid impulse edge; the signal after the A/D conversion in the controller was transmitted to the data processing system in the computer via Ethernet. In accordance with the extracted feature points, the required parameters were calculated, the results were communicated to the CNC system through RS232 serial ports and the unqualified workpiece was corrected by turning, thus completing the whole closed loop process of on-machine measurement. Figure 12 describes a signal flowchart of the on-machine measurement experiment.

**Figure 13:**
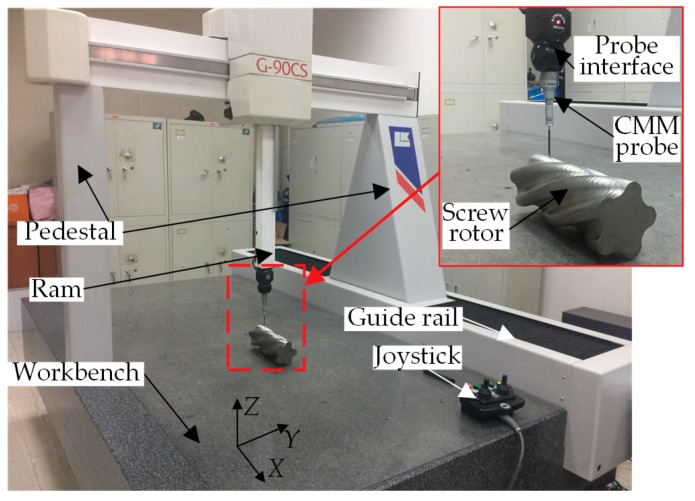
Comparative experiment for measuring the screw rotor with CMM.

To verify the application effect of the proposed LT data acquisition method in spiral curved surface measurement, firstly, the axial section of the measured screw rotor was marked and the measurement position and measurement phase of the mark were recorded. Then, the comparative measurement experiment for the same screw along the same path was performed by using CMM and MPG. The CMM measurement experiment is shown in Figure 13. The technical parameters and measurement processes of CMM were the same as those for API threads, and the special spiral curved surface measurement software was also used, with no details described here anymore. The results of the screw rotor parameters obtained by the three measurement methods along the same measurement path are shown in Table 3. The grouping of results obtained by various measurement methods was the same as that in Table 2. Moreover, the results in Table 3 are all the average of 10 measurements, and the nominal value and tolerance zone of each parameter of the 165-type screw rotor are also given in the table.

To ensure the integrity of measurement results by OMM 3, it is also necessary to evaluate the measurement uncertainty of the screw rotor’s major diameter *d*_1_, minor diameter *d*_2_ and pitch *w*. The main factors affecting the measurement uncertainty of major diameter *d*_1_ and minor diameter *d*_2_ are LDS resolution and linearity errors, environmental temperature changes and measurement repeatability. Furthermore, the main factors affecting the measurement uncertainty of pitch *w* are LDS resolution, *Z*-axial positioning accuracy of the machine tool, straightness of the axial rail, environmental temperature changes and measurement repeatability. In accordance with the method for evaluating the measurement uncertainty of thread parameters, the standard deviation of 10 measurements of the screw rotor’s major diameter *d*_1_ was 0.0029 μm, and the combined uncertainty *u_d_*_1_ = 1.80 μm; the standard deviation of 10 measurements of the screw rotor’s minor diameter *d*_2_ was 0.0032 μm, and the combined uncertainty *u_d_*_2_ = 1.79 μm; the standard deviation of 10 measurements of the screw rotor’s pitch *w* was 0.0012 μm, and the combined uncertainty *u_w_* = 4.65 μm. Furthermore, the uncertainty values of each screw rotor parameter obtained by using OMM 3 are likewise listed in Table 3.

## 4. Discussions

The straight comparison of the two sets of data in Figure 8 illustrates that the data acquired by the traditional GG method greatly deviate from the actual contour due to the influence of spot edge noise area. However, in the improved algorithm, the gradient threshold method and Gaussian fit algorithm are used to exactly eliminate the interference of the spot edge noise area. Concurrently, polynomial interpolation is used to enhance the data density of the traditional GG method, and the acquired data are greatly closer to the real situation. The thread height calculation results acquired by the above two algorithms are compared with CMM results, of which the thread height error in Figure 8a is 0.016 mm and the thread height error in Figure 8b is 0.008 mm. The comparison results disclose that the data acquisition accuracy of the variable threshold sub-pixel GG extraction algorithm proposed in this paper outperforms that of the traditional GG method. Additionally, the analysis results of the data in Table 2 show that the differences are all within the tolerance zone, which indicates that the API thread processing is qualified. Compared with the measurement accuracy of OMM 1, OMM 2 and OMM 3, the variable threshold GG extraction algorithm and the image sensor inclination error model can increase the measurement accuracy of free-form curved surfaces using LT. Through the comparison of the results by OMM 3 and by MPG, the measurement accuracy of OMM 3 proposed in this paper is higher than that of the traditional MPG, and the former reduces the measurement time and also increases the measurement accuracy of API threads. The comparison of the results by on-machine measurement and by CMM also shows that the API thread measurement results obtained by using the proposed LT data acquisition method are even closer to those of the CMM with higher measurement accuracy. Additionally, the measurement uncertainty of OMM 3 for each parameter of the NC50 API thread is 0.51 μm for thread height, 0.40 μm for pitch and 2.76′ for half of the thread angle, respectively. It should be especially explained that the above uncertainty values are the repeatability indicators of the thread measuring system using OMM 3, and they are not complete or comprehensive. During the measurement, the sensor feed speed *F* = 200 mm/min and the measured thread is 11 mm long, and then, the one-trial measurement time by using OMM 3 is only 7.2 s.

Through the comparison of the parameters acquired by the three measurement methods with the corresponding nominal values, it can be concluded that the differences are all within the tolerance zone, which indicates that the measured screw rotor is qualified. Compared with the measurement accuracy of OMM 1 and OMM 2, the proposed image sensor inclination error model can increase the measurement accuracy of free-form curved surfaces using LT. Further, compared with the measurement accuracy of OMM 1, OMM 2 and OMM 3, the variable threshold sub-pixel GG center extraction algorithm highly improves the LT data acquisition accuracy in free-form curved surface measurement. Through the comparison of the data acquired by OMM 3 and MPG, the measurement accuracy of the on-machine measurement method proposed in this paper is higher than that of the traditional MPG, and the former saves the measurement time and also improves the measurement accuracy by 70%. The comparison of the results by OMM and by CMM also shows that the screw rotor measurement results obtained by using the proposed LT data acquisition method are even closer to those of the CMM with higher measurement accuracy. Additionally, the measurement uncertainty of OMM 3 for each parameter of the 165-type screw rotor is 1.80 μm for major diameter, 1.79 μm for minor diameter and 4.65 μm for pitch, respectively. Similarly, the above uncertainty values are the repeatability indicators of the screw rotor measuring system using OMM 3, and they are also not complete or comprehensive. As the sensor feed speed is *F* = 200 mm/min, the measuring length is 140 mm and the spindle rotation speed is 10 r/min, it spends 52.1 s in using OMM 3 to conduct one-trial measurement of one screw rotor pitch, which meets the requirements of rapid measurement of large-size workpieces in online production.

Judging from the standard deviation of 10 measurements of each parameter, it can be seen that the variable threshold sub-pixel GG extraction algorithm has realized high-accuracy acquisition of laser spots, effectively controlled the dispersion of measurement results and better ensured the measuring stability. Additionally, through the comparison of the data in Table 2 and Table 3 it can also be found that because the image sensor inclination error model is only affected by the measuring distance, when the LDS is used to measure two kinds of free-form curved surfaces, the amount of compensation for the two by the model is 2 μm for both. However, in terms of the improvement of the data acquisition accuracy, there is a big difference between the thread curved surface and the spiral curved surface by using the variable threshold sub-pixel GG extraction algorithm. The measurement accuracy of API thread parameters by using the method is improved by 2 μm, while that of screw rotor parameters is improved by 8 μm. The reason for the resulting difference is that compared with the spiral curved surfaces, the thread curved surface is more closed and more intense in fluctuation. Consequently, the spot information received by the image sensor is incomplete, thus hampering the spot center extraction accuracy. Therefore, the improved spot center extraction algorithm proposed in this paper is more suitable for the measurement of free-form curved surfaces with smaller curvature and more uniform curvature changes.

## 5. Conclusions

To increase the data acquisition accuracy of LT, this paper starts with the LT principle, delves into the LT geometrical optical path system, analyzes the cause for generating image sensor inclination errors and deduces a mathematical model that can quantitatively calculate the error compensation amount based on the deviation of the spot centroid. Through the established model, the influence law of the measuring distance on the data acquisition accuracy of LT is achieved. The research results are successfully applied to measurement of free-form curved surfaces. Further, the variable threshold sub-pixel GG extraction algorithm is proposed. The gradient function and Gaussian fit algorithm are used to set thresholds to remove the interference of spot edge noise in the traditional GG method. Additionally, polynomial interpolation is used to increase the spot sub-pixel point density, thus improving the extraction accuracy of the spot center using LT. Furthermore, the above research results are used in on-machine measurement of API threads and screw rotors, respectively. The measurement results prove that the image sensor inclination error model is effectively accurate and has the identical effect of compensating the collected data of free-form curved surfaces in different states. The improved spot center extraction algorithm can boost the data acquisition accuracy of LT and be better applied to measurement of free-form curved surfaces with smaller curvature and more uniform curvature changes. To sum up, the combination of the said two can greatly improve the measurement accuracy of free-form curved surfaces using LT.

## Figures and Tables

**Table 1:** Main parameters of the KEYENCE laser displacement sensor (LDS).

Item	Specifications
Model No.	LKH080
Measurement center distance	80 mm
Measuring range	±18 mm
Repeatability	0.1 μm
Resolution	0.1 μm
Linearity	±0.02% FS (full scale, FS)
Sampling frequency	1 KHz
Spot size	200 × 750 μm
Communication mode	RS232 (recommend standard 232, RS232) and Ethernet

**Table 2:** Comparison of the three measuring methods for API threads. CMM, coordinate measuring machine; MPG, monomial parameters gauge; OMM, on-machine measurement.

	CMM	MPG	OMM 1	OMM 2	OMM 3	Uncertainty	Nominal Values
*p*/mm	6.345	6.354	6.355	6.353	6.350	0.51 × 10^−3^	6.$35_{-0.01}^{+0.01}$
*h*/mm	3.126	3.116	3.116	3.118	3.120	0.40 × 10^−3^	3.$095_{0}^{+0.12}$
*α*/2	30°02′		29°38′	29°46′	29°56′	2.76′	${30{}^{\circ}}_{-45\prime }^{+45\prime }$

**Table 3:** Comparison of the three measuring methods for screw rotors.

	CMM	MPG	OMM 1	OMM 2	OMM 3	Uncertainty	Nominal Values
*d*_1_/mm	98.012	97.995	97.995	97.997	98.006	1.80 × 10^−3^	$98_{-0.05}^{+0.05}$
*d*_2_/mm	66.988	67.005	67.003	67.001	66.994	1.79 × 10^−3^	$67_{-0.05}^{+0.05}$
*w*/mm	130.005	130.012	130.012	130.010	130.005	4.65 × 10^−3^	$130_{-0.01}^{+0.01}$
